# Early Pregnancy Regulates Expression of IkappaB Family in Ovine Spleen and Lymph Nodes

**DOI:** 10.3390/ijms24065156

**Published:** 2023-03-08

**Authors:** Shengya Fang, Chunjiang Cai, Ying Bai, Leying Zhang, Ling Yang

**Affiliations:** School of Life Sciences and Food Engineering, Hebei University of Engineering, Handan 056038, China

**Keywords:** IkappaB, lymph node, pregnancy, spleen, sheep

## Abstract

Early pregnancy modulates the maternal immune system, including the spleen and lymph nodes, which participate in maternal innate and adaptive immune responses. Methods: Ovine spleens and lymph nodes were sampled at day 16 of the estrous cycle, and at days 13, 16 and 25 of gestation, and qRT-PCR, Western blot and immunohistochemistry analysis were used to analyze the expression of the IκB family, including BCL-3, IκBα, IκBβ, IκBε, IKKγ, IκBNS and IκBζ. Early pregnancy induced expression of BCL-3, IκBα, IκBε, IKKγ and IκBζ, and expression of BCL-3, IκBβ and IκBNS peaked at day 16 of pregnancy in the spleen. However, early pregnancy suppressed the expression of BCL-3 and IκBNS, but stimulated the expression of IκBβ and IκBζ, and expression levels of IκBα, IκBβ, IκBε and IKKγ peaked in lymph nodes at days 13 and/or 16 of pregnancy. Early pregnancy changed the expression of the IκB family in the maternal spleen and lymph node in a tissue-specific manner, suggesting that the modulation of the IκB family may be involved in regulation of maternal functions of the spleen and lymph nodes, which are necessary for the establishment of maternal immune tolerance during early pregnancy in sheep.

## 1. Introduction

The relationship between mother and fetus is an immunological marvel and enigma, as averting maternal–fetal conflict is continuously required throughout pregnancy [1]. There are significant changes in maternal immune system components that interact with the fetal immune system dynamically and co-operatively to contribute to the modulation of the risk of infection and the course of immunological disease during pregnancy [2]. Ruminant conceptus signaling (interferon-tau, IFNT) modulates the innate immune system in paracrine and endocrine manners, which is essential for successful pregnancy establishment and prevention of rejection against the allogenic conceptus by the mother [3]. The immune system changes in response to the presence of an embryo, and these responses are different in local and peripheral immune tissues during early pregnancy in sheep [4]. Nuclear factor kappa B (NF-κB) is implicated in both physiological and pathological processes; NF-κB regulation is essential during pregnancy, and dysregulation results in premature termination of pregnancy, with bad outcomes for the mother and the fetus [5]. Early pregnancy modulates the expression of NF-κB components in the maternal thymus, spleen, liver and inguinal lymph nodes, which is necessary for embryo implantation and pregnancy maintenance in sheep [6,7,8,9].

The activities of NF-κB proteins are strictly repressed by inhibitors of NF-κB (IκB) proteins, including IκBα, IκBβ, IκBε, IKKγ, B cell leukemia-3 (BCL-3), IκBNS (also known as NFKBID) and IκBζ, and IκBβ plays a crucial role in the regulation of innate immune responses [10]. BCL-3 can either promote or inhibit NF-κB target gene expression, which is dependent on the type of cell, the type of activating stimulus and the type of the NF-κB target gene involved [11]. IκB proteins not only interact with NF-κB to change its transcriptional activity, but also bind to chromatin and control gene expression to participate in immune homeostasis [12]. BaelanChagsangBang (BCB) water extract has strong anti-oxidative and cytoprotective effects in vitro, and in vivo administration of BCB water extract can improve the number of implantation sites in pregnant mice through upregulation of IκBα expression [13]. IKKβ signaling is involved in the prevention of preterm delivery and the improvement of neonatal outcomes through the suppression of production of interleukin-6 (IL-6) and related cytokines [14]. IκB proteins are related to successful pregnancy.

The spleen plays a key role in adaptive immune responses, and the splenic nerve is involved in modulation of the central amygdala and the paraventricular nucleus corticotrophin-releasing hormone-producing neurons [15]. OLT1177 (an orally active β-sulfonyl nitrile molecule) inhibits activation of the nucleotide-binding oligomerization domain (NOD)-like receptor protein 3 inflammasome, which results in a downregulation of phosphorylated NF-κB and IκB kinase levels in spleen cells from OLT1177-treated mice [16]. There is a differential expression of costimulatory molecules on splenic antigen-presenting cells in spleens of female mice during the preimplantation period of pregnancy, which are related to pregnancy outcome and the tolerogenic immune response in mice [17].

Lymph nodes are involved in adaptive immunity through modulating T cell and B cell activation and their differentiation into effector cells [18]. There is a downregulation of the specific alloreactivity during the pre-implantation and implantation stages of pregnancy, but the specific and non-specific alloreactivities are upregulated at mid-pregnancy in para-aortic lymph node cells [19]. The immune functions of the lymph nodes around the reproductive tract and other peripheral lymph nodes are different during pregnancy, which are involved in the modulation of the maternal immune system in ewes [4]. Lymphatic endothelial cells modulate progesterone bioavailability, which is essential for regulating immune tolerance during pregnancy in humans [20].

Previous studies report that interferon-stimulated genes (ISGs), progesterone receptor, progesterone-induced blocking factor, tumor necrosis factor (TNF)-β, IL-2, IL-5, IL-6, IL-10, cyclooxygenase 2, aldo-keto reductase family 1, member B1, melatonin receptor 1 (MT1), gonadotropin-releasing hormone and its receptor are upregulated, but MT2 is downregulated in the maternal spleen of ewes during early pregnancy [21,22,23,24,25,26]. In addition, early pregnancy modulates the expression of Toll-like receptor signaling members and NF-κB components in the ovine maternal spleen [7,27]. Furthermore, the expression of ISGs, progesterone receptor, prostaglandin synthases, Th cytokines, MT1, gonadotropin-releasing hormone and its receptor, and prolactin and its receptor are changed in lymph nodes during early pregnancy in sheep [24,25,28,29,30,31,32]. On the other hand, early pregnancy regulates the expression of Toll-like receptor signaling members, complement components, nuclear factor kappa B family and NOD receptors in the ovine maternal lymph nodes [9,33,34,35].

It is hypothetic that early pregnancy modulates the expression of IκB proteins in the ovine spleen and inguinal lymph nodes. The aim of this study was to analyze the expression of BCL-3, IκBα (NFKBIA), IκBβ (NFKBIB), IκBε (NFKBIE), IKKγ (IKBKG), IκBNS (NFKBID) and IκBζ (NFKBIZ) in the maternal spleen and lymph nodes of sheep, which may be beneficial for understanding the maternal immunomodulation of spleen and lymph nodes during early pregnancy in sheep.

## 2. Results

### 2.1. Expression of BCL-3, NFKBIA, NFKBIB, NFKBIE, IKBKG, NFKBID and NFKBIZ mRNA in the Spleen and Lymph Nodes

**Figure 1:**
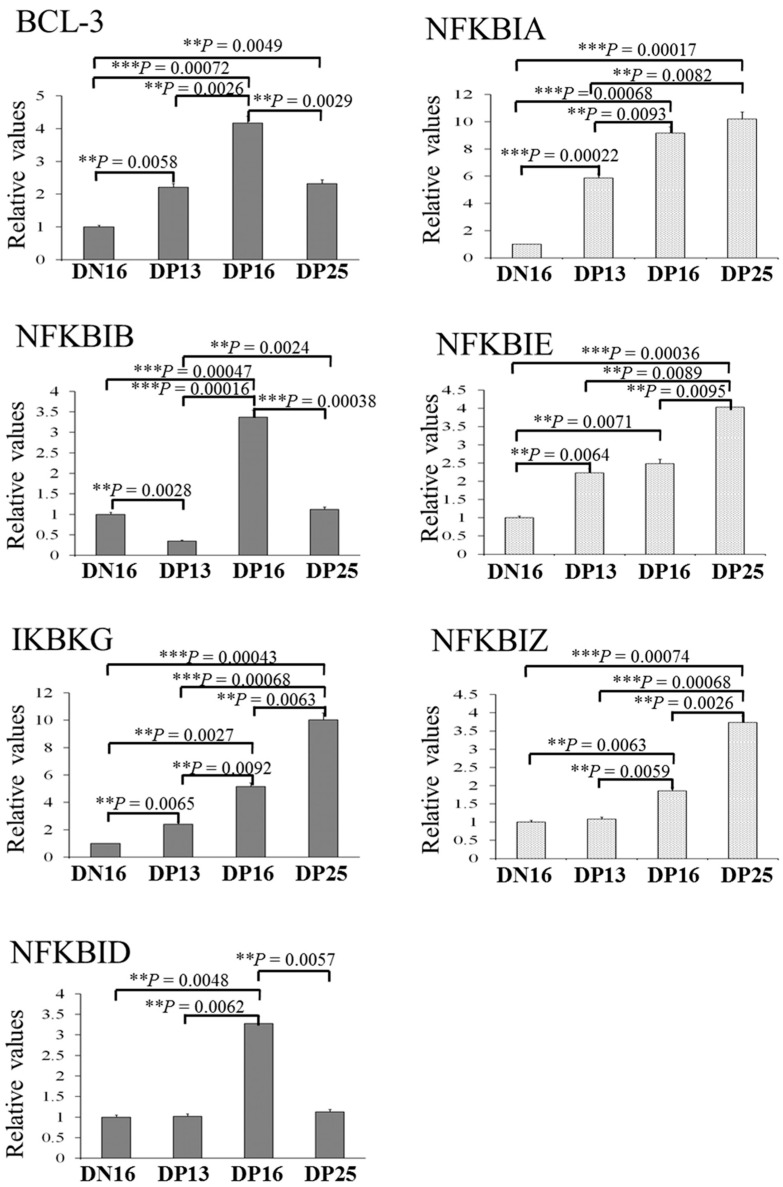
Relative expression values of *BCL-3*, *NFKBIA*, *NFKBIB*, *NFKBIE*, *IKBKG*, *NFKBID* and NFKBIZ mRNA in ovine spleen measured by quantitative real-time PCR. Note: DN16 = day 16 of the estrous cycle; DP13 = day 13 of pregnancy; DP16 = day 16 of pregnancy; DP25 = day 25 of pregnancy. Significant differences (*p* < 0.05) are indicated by different letters within same color columns. *p* values between 0.001 and 0.01 are shown with two (**) asterisks, and *p* value less than 0.001 is designated with three (***) asterisks.

Figure 1 showed that the relative expression level of *BCL-3* mRNA was increased in the spleen during early pregnancy compared to day 16 of the estrous cycle, with a peak at day 16 of pregnancy (*p* < 0.05). The relative expression levels of *NFKBIA*, *NFKBIE*, *IKBKG* and *NFKBIZ* mRNA gradually increased from day 13 to 25 of pregnancy (*p* < 0.05). Furthermore, there was a peak in the relative expression levels of *NFKBIB* and *NFKBID* mRNA at day 16 of pregnancy, but the expression level of *NFKBIB* mRNA was the lowest at day 13 of pregnancy (*p* < 0.05).

**Figure 2:**
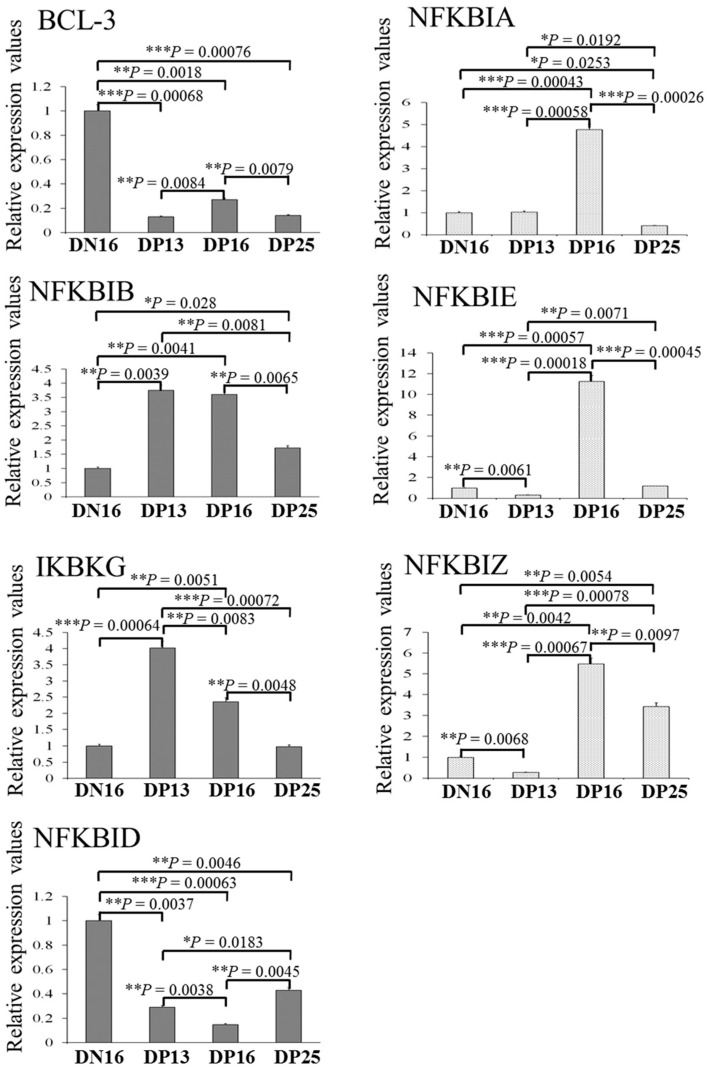
Relative expression values of *BCL-3*, *NFKBIA*, *NFKBIB*, *NFKBIE*, *IKBKG*, *NFKBID* and *NFKBIZ* mRNA in the lymph nodes from non-pregnant ewes and pregnant ewes. Note: DN16 = day 16 of the estrous cycle; DP13 = day 13 of pregnancy; DP16 = day 16 of pregnancy; DP25 = day 25 of pregnancy. Significant differences (*p* < 0.05) are indicated by different letters within same color columns. *p* values between 0.01 and 0.05 are shown with one (*) asterisk, and *p* values between 0.001 and 0.01 are shown with two (**) asterisks, and *p* value less than 0.001 is designated with three (***) asterisks.

In the lymph nodes, early pregnancy suppressed the expression of *BCL-3* and *NFKBID* mRNA, but stimulated the expression of *NFKBIB* and *NFKBIZ* mRNA at days 16 and 25 of pregnancy (*p* < 0.05; Figure 2). In addition, there was a peak in the relative expression levels of *NFKBIB* and *IKBKG* mRNA at days 13 and 16 of pregnancy (*p* < 0.05). Furthermore, expression levels of *NFKBIA*, *NFKBIE* and *NFKBIZ* mRNA peaked at day 16 of pregnancy, but expression level of *NFKBIA* was the lowest at day 25 of pregnancy (*p* < 0.05), and expression levels of *NFKBIE* and *NFKBIZ* mRNA were the lowest at day 13 of pregnancy among the four groups (*p* < 0.05).

### 2.2. Expression of IκB Proteins in the Spleen and Lymph Nodes

**Figure 3:**
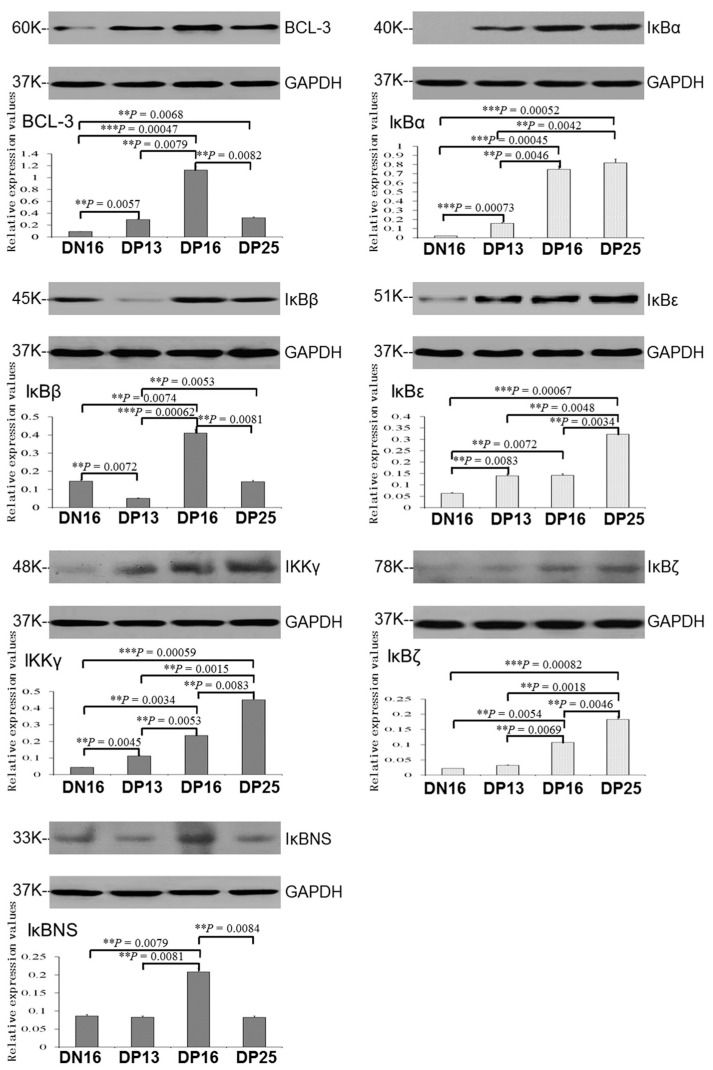
Expression of IκB family proteins in ovine spleen analyzed by Western blotting. Note: DN16 = day 16 of the estrous cycle; DP13 = day 13 of pregnancy; DP16 = day 16 of pregnancy; DP25 = day 25 of pregnancy. Significant differences (*p* < 0.05) are indicated by different superscript letters within the same color column. *p* values between 0.001 and 0.01 are shown with two (**) asterisks, and *p* value less than 0.001 is designated with three (***) asterisks.

It was revealed in Figure 3 that there was an upregulation of BCL-3 protein during early pregnancy compared to day 16 of the estrous cycle, and the expression level of IκBβ protein was the highest at day 16 of pregnancy (*p* < 0.05). Early pregnancy induced gradual upregulation of IκBα, IκBε, IKKγ and IκBζ proteins from day 13 to 25 of pregnancy in the spleen (*p* < 0.05), and IκBα, IKKγ and IκBζ proteins were almost undetected at day 16 of the estrous cycle. In addition, expression of IκBβ and IκBNS proteins was the highest at day 16 of pregnancy (*p* < 0.05), but IκBβ protein was almost undetected at day 13 of pregnancy.

**Figure 4:**
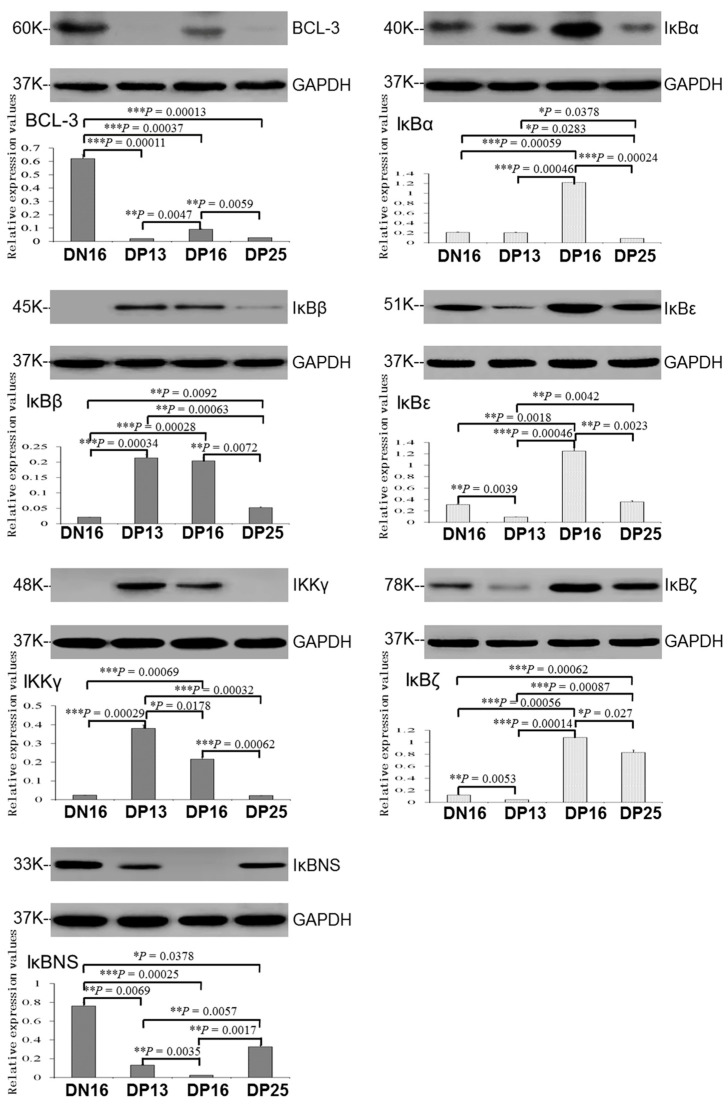
Expression of IκB family proteins in lymph nodes from non-pregnant ewes and pregnant ewes. Note: DN16 = day 16 of the estrous cycle; DP13 = day 13 of pregnancy; DP16 = day 16 of pregnancy; DP25 = day 25 of pregnancy. Significant differences (*p* < 0.05) are indicated by different letters within the same color column. *p* values between 0.01 and 0.05 are shown with one (*) asterisk, and *p* values between 0.001 and 0.01 are shown with two (**) asterisks, and *p* value less than 0.001 is designated with three (***) asterisks.

In the lymph nodes, expression of BCL-3 and IκBNS proteins was downregulated during early pregnancy (*p* < 0.05; Figure 4), and BCL-3 protein was almost undetected at days 13 and 25 of pregnancy, while IκBNS protein was almost undetected at day 16 of pregnancy. In addition, expression levels of IκBβ and IKKγ proteins peaked at days 13 and 16 of pregnancy (*p* < 0.05), IκBβ protein was almost undetected at day 16 of the estrous cycle and IKKγ protein was almost undetected at day 16 of the estrous cycle and day 25 of pregnancy. Furthermore, expression levels of IκBα, IκBε and IκBζ proteins peaked at day 16 of pregnancy, but IκBα protein level was the lowest at day 25 of pregnancy (*p* < 0.05), and levels of IκBε and IκBζ proteins were the lowest at day 13 of pregnancy among the four groups (*p* < 0.05).

### 2.3. Immunohistochemistry for IκBβ and IKKγ Proteins in the Spleen and Lymph Nodes

**Figure 5:**
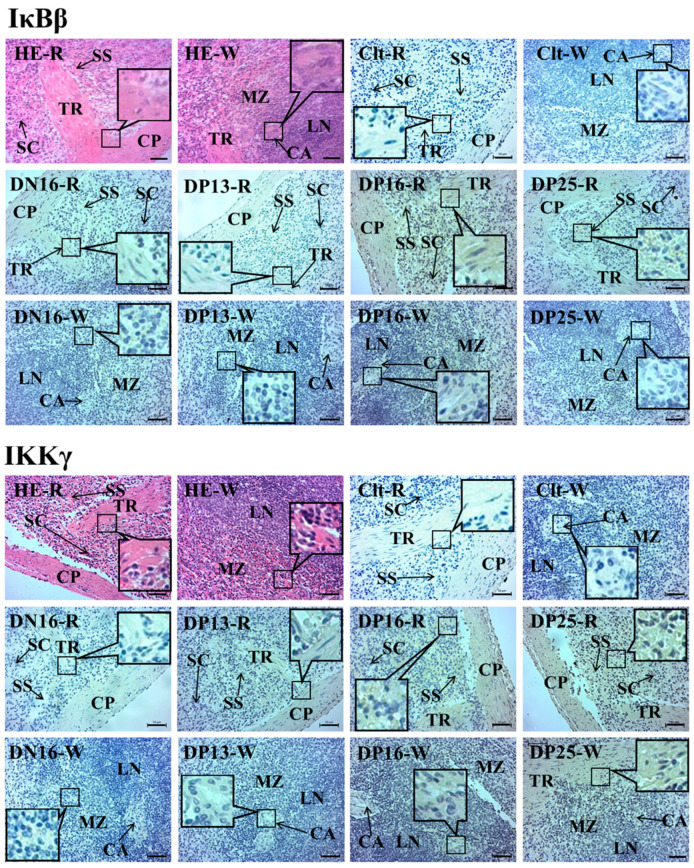
Representative immunohistochemical localization of IκBβ and IKKγ proteins in ovine spleen. The spleen is divided into red pulp (R) and white pulp (W), and surrounded by a thickened capsule. Capsule (CP) with several trabeculae (TR) projects into the substance of the spleen. Note: HE = stained by hematoxylin and eosin; Clt = negative control; SS = splenic sinuses; SC = splenic cords; MZ = marginal zone; LN = lymphoid nodule; CA = central arteriole; DN16 = day 16 of the estrous cycle; DP13 = day 13 of pregnancy; DP16 = day 16 of pregnancy; DP25 = day 25 of pregnancy. Bar = 50 µm.

In the spleen, IκBβ and IKKγ proteins were located in the capsule, trabeculae and splenic cords. For the negative control, the spleens from day 16 of the estrous cycle, and spleens from days 13, 16, and 25 of pregnancy, the staining intensities for IκBβ protein were 0, 1, 0, 3 and 1, and the staining intensities for IKKγ protein were 0, 0, 1, 2 and 2, respectively (Figure 5). The staining intensity was as follows: 0 = negative; 1 = weak; 2 = strong; 3 = stronger.

**Figure 6:**
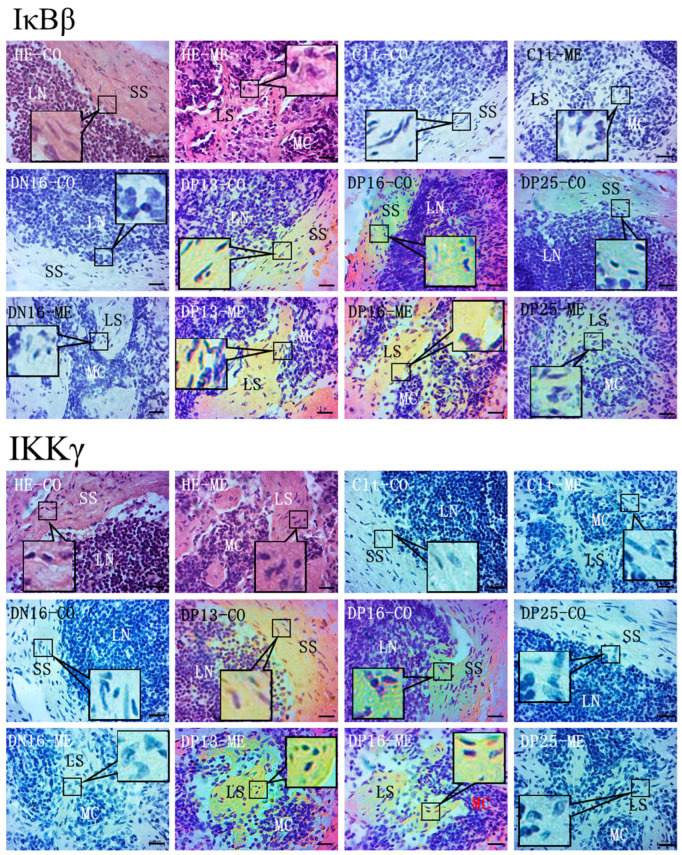
Representative immunohistochemical localization of IκBβ and IKKγ proteins in the lymph nodes from non-pregnant ewes and pregnant ewes. The lymph node is divided into the outer cortex (CO) and the inner medulla (ME). Lymph enters the convex through the subcapsular sinus (SS) around the lymphoid nodules (LN), and flows into the medulla through the lymph sinus (LS) around the medullary cord (MC). Note: HE = stained by hematoxylin and eosin; Clt = negative control; DN16 = day 16 of the estrous cycle; DP13 = day 13 of pregnancy; DP16 = day 16 of pregnancy; DP25 = day 25 of pregnancy. Bar = 20 µm.

In lymph nodes, IκBβ and IKKγ proteins were limited to the subcapsular sinus and lymph sinuses, but there was almost no immunostaining in the lymphoid nodule and medullary cords (Figure 6). The staining intensities for IκBβ protein were 0, 0, 3, 3 and 1, and the staining intensities for IKKγ protein were 0, 0, 3, 2 and 0, for the negative control, the lymph nodes from day 16 of the estrous cycle and lymph nodes from days 13, 16 and 25 of pregnancy, respectively.

## 3. Discussion

BCL-3 is involved in the immune response through interactions with the NF-κB subunits, and plays essential roles in germinal center formation and marginal zone B-cell development [36,37]. There is an upregulation of NF-κB1 or NF-κB2 in the maternal spleen during early pregnancy [7], suggesting that BCL-3 participates in the regulation of adaptive immunity through binding homodimers of NF-κB1 or NF-κB2. BCL-3 is required for dendritic cells in effective priming of CD4 and CD8 T cells and generates adaptive immunity in mice [38]. However, BCL-3 is upregulated in human placentas of severe early onset pre-eclampsia cases, which is related to immunology functions [39]. In addition, there is an upregulation of BCL-3 in lymphoid malignancy, and BCL-3-transgenic mice show splenomegaly and an accumulation of mature B cells in lymph nodes [40]. In this study, early pregnancy induced upregulation of *BCL-3* mRNA and protein in the maternal spleen, but inhibited expression of BCL-3 in the maternal lymph nodes. Therefore, early pregnancy induced tissue-specific expression of BCL-3 in the maternal spleen and lymph nodes. The upregulation of BCL-3 in the maternal spleen may be associated with modulation of splenic adaptive immunity, but the downregulation of BCL-3 in maternal lymph nodes may be related to immune tolerance.

Circulating fetal DNA activates NF-κB and degrades IκBα in human peripheral blood mononuclear cells (PBMCs), resulting in the production of proinflammatory cytokines, which is related to spontaneous preterm birth [41]. IκBα plays key roles in proper B cell and secondary lymphoid tissue formation, which is related to NF-κB activation potentials in the mature B cells of mice [42]. IκBα level in cytoplasmic fractions from PBMCs of pregnant females is decreased compared with nonpregnant women, but this is not related to NF-κB activation in pregnancy [43]. In addition, IκBα level is downregulated in the lymph nodes of mice afflicted with murine-acquired immunodeficiency syndrome, but this does not lead to upregulation of *NF-κB* DNA binding activity [44]. Our results revealed that early pregnancy stimulates expression of IκBα in the maternal spleen, and IκBα level in the maternal lymph node peaked at day 16 of pregnancy, but declined at day 25 of pregnancy. Therefore, the upregulation of IκBα in the maternal spleen may be necessary for maturation of splenic B cells and pregnancy maintenance, and changed expression of IκBα in the maternal lymph node may be related to maternal immunoregulation during early pregnancy in sheep.

The E3 ligase ARIH2 causes degradation of IκBβ in the nucleus of dendritic cells, which is essential for embryogenesis [45]. NF-κB activity is suppressed in PBMCs from pregnant females, and IκBβ level is decreased more in pre-eclampsia [43]. It was revealed in this study that *IκBβ* mRNA and protein levels were declined on day 13 of pregnancy, but upregulated on day 16 of pregnancy in the maternal spleen, and IκBβ protein was located in the capsule, trabeculae and splenic cords. However, pregnancy stimulated the expression of IκBβ in the maternal lymph nodes, with peaks at days 13 and 16 of pregnancy, and IκBβ protein was limited to the subcapsular sinus and lymph sinuses. Therefore, the changing expression of IκBβ in the maternal spleen and lymph nodes in a tissue-specific manner may be involved in the immune regulation of maternal spleen and lymph nodes in sheep.

TNF-α and insulin-like growth factor-I cause pathological changes of placentas, and lead to pre-eclampsia, changing NFKBIE DNA methylation in BeWo cells [46]. As a negative regulator of the transcription factor NF-κB, IκBε interacts with RelA- and cRel-specific dimers to regulate B cell proliferation and survival in a stimulus-specific manner [47]. *IκBε* deletion results in increased lymph node cellularity and enhanced basal thymidine incorporation by lymphoid cells ex vivo, as well as enhancing the survival of ex vivo splenic B cells, which are regulated via c-Rel-dependent lymphoid responses in murine T- and B cells [48]. IκBε is mainly expressed in T cells in the spleen and lymph nodes, and IκBε deletion results in the reduction of one T cell precursor subspecies in mice, but does not lead to observable augmentation of constitutive nuclear NF-κB/Rel-binding activity [49]. It was found in this study that expression levels of IκBε and IκBα were upregulated in the maternal spleen during early pregnancy. However, the expression level of IκBε was the lowest at day 13 of pregnancy, but the highest at day 16 of pregnancy in the maternal lymph nodes. Therefore, the upregulation in the spleen and changed expression of IκBε in the lymph nodes may participate in regulating the immune functions of the maternal spleen and lymph nodes during early pregnancy in sheep.

NF-κB essential modulator (NEMO), also known as IκB kinase γ (IKKγ), plays a central role in the innate immune system by regulating the IKKα-IKKβ signaling axis [50]. *NEMO* gene expression level in maternal blood is higher, but lower in placentas of women with pre-eclampsia than healthy controls, suggesting that *NEMO* gene expression is associated with pre-eclampsia development in a tissue-specific manner [51]. *NEMO* mutation causes immune deficiency, which impairs lymph node formation in hemizygous mice and men [52]. In addition, *NEMO* knockout in T-lymphocyte induces the expression of Th17-related cytokines in spleen CD4+ T cells, and interrupted the canonical NF-κB pathway in an experimental nephrotoxic nephritis mouse model [53]. Our data revealed that *IKKγ* mRNA and protein levels gradually upregulated from day 13 to 25 of pregnancy in the maternal spleen, and IKKγ protein was located in the capsule, trabeculae and splenic cords. However, *IKKγ* mRNA and protein levels peaked at days 13 and 16 of pregnancy in the maternal lymph nodes, and IKKγ protein was limited to the subcapsular sinus and lymph sinuses. Therefore, the changed tissue-specific expression of IKKγ in maternal spleen and lymph nodes may be involved in the immune regulation of maternal spleen and lymph nodes.

IκBζ has six ankyrin repeats that are conserved in other IκB proteins, and its mRNA expression is induced rapidly following lipopolysaccharide injection in the spleen of mice [54]. IκBζ induces IL-17 production by helper T cells, which is critical for the proliferation of lymph node and splenic stromal cells [55]. IκBζ negatively modulates NF-κB activation in lymph nodes, which is beneficial for inhibiting the transformation and development process of lymphomas [56]. Our results showed that expression of IκBζ mRNA and protein increased at days 16 and 25 of pregnancy in the maternal spleen and lymph nodes. Therefore, the upregulation of IκBζ may be related to negative modulation of NF-κB activation, which may contribute to the immune tolerance of the maternal spleen and lymph nodes during early pregnancy.

IκBNS is a negative nuclear regulator of NF-κB activity that regulates IL-6 and TNF-α transcription, which are important factors in the remodeling of the uterus for blastocyst implantation in mice [57]. IκBNS negatively regulates IL-6 production under transcriptional control of NF-κB that plays an essential role in embryo implantation and the onset of labor during pregnancy in mice [58]. PBMCs in miscarrying women produce significantly higher concentrations of Th1 cytokines compared with normal pregnancy, which indicates that Th1 cytokines are deleterious to successful pregnancy in humans [59]. *IκBNS* knock-out results in a B cell extrinsic defect in the spleen and inguinal lymph node in mice [60]. Our data revealed that *IκBNS* mRNA and protein levels peaked at day 16 of pregnancy in the maternal spleen, but IκBNS expression level was the lowest at day 16 of pregnancy in the maternal lymph nodes. Therefore, the peak in the maternal spleen and the lowest level of IκBNS in lymph nodes at day 16 of pregnancy may be involved in the modulation of the maternal functions of the spleen and lymph nodes in a tissue-specific manner during early pregnancy in sheep.

**Figure 7:**
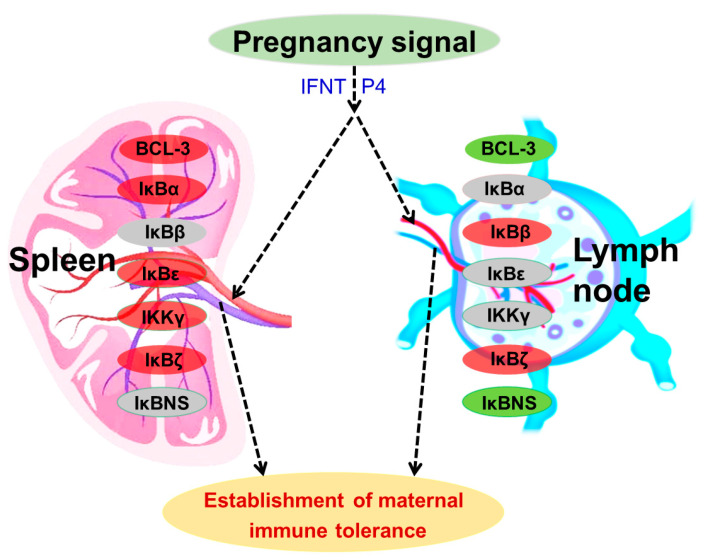
Proposed sketch of IκB family in maternal spleen and lymph nodes during early pregnancy in sheep. Early pregnancy signals, including interferon-tau (IFNT) and progesterone (P4), induce changed expression of IκB family, including B cell leukemia-3 (BCL-3), IκBα, IκBβ, IκBε, IKKγ, IκBζ and IκBNS, in a tissue-specific manner, which contributes to establishment of maternal immune tolerance during early pregnancy. Note: red, stimulators; green, negative regulators; blue, changed.

Type I interferons, including IFNT, induce hundreds of through activations of a number of cellular factors, including the IκB kinase epsilon [61]. There is a negative interaction between NF-κB and progesterone receptor [62]. In addition, our previous studies reported that ISGs and progesterone receptor are upregulated in ovine lymph nodes and spleen during early pregnancy, which are related to maternal immune tolerance [21,23,26,28,29,31]. During early pregnancy in sheep, early pregnancy signals (IFNT and progesterone) exert effects on the expression of the IκB family in the maternal spleen and lymph nodes, which upregulate the expression of BCL-3, IκBα, IκBε, IKKγ and IκBζ in the spleen, and IκBβ and IκBζ in the lymph nodes. However, early pregnancy signals inhibit the expression of BCL-3 and IκBNS in the lymph nodes, and also modulate the expression of IκBβ and IκBNS in the spleen and IκBα and IKKγ in the lymph nodes. Therefore, early pregnancy modulates the expression of the IκB family in the maternal spleen and lymph nodes in a tissue-specific manner, which may contribute to the establishment of maternal immune tolerance during early pregnancy (Figure 7).

## 4. Materials and Methods

### 4.1. Animal Tissue Collection

All procedures were approved by the Hebei University of Engineering Animal Care and Use Committee (application number 2019-017). Mature Small-tail Han ewes (approximately 18 month old) were purchased from Handan Boyuan Animal Husbandry Co., Ltd., Handan (China), and housed using conventional breeding and nutrition. The ewes with normal estrous cycles were randomly divided into three groups of pregnant ewes (days 13, 16 and 25 of pregnancy) and a group of day 16 of the estrous cycle (*n* = 6 for each group). After detection of sexual receptivity (day 0 of pregnancy or nonpregnancy) with a vasectomized ram, for the three groups, ewes were bred with intact rams, and the nonpregnant ewes were not mated with an intact ram. Spleens and inguinal lymph nodes were sampled on days 13, 16 and 25 post-estrus after the females were killed. Pregnancy was confirmed through sighting a conceptus in the uterus. Samples of spleens and lymph nodes were immediately fixed in fresh 4% (*w*/*v*) paraformaldehyde, and also frozen, stored for following quantitative real-time PCR (qRT-PCR) and Western blot analysis.

### 4.2. RNA Extraction and qRT-PCR Assay

Total RNA was isolated from the samples of spleens and lymph nodes following the manufacturer’s instructions using TRNzol reagent (Tiangen Biotech Co., Ltd., Beijing, China), and a Nanodrop spectrophotometer (Thermo Fisher Scientific, Wilmington, NC, USA) was applied to measure the concentration of total RNA at 260/280 nm. The cDNA was synthesized according to a FastQuant RT kit with DNase (Tiangen Biotech Co., Ltd., Beijing, China) protocol. The mRNA expression values of *BCL-3*, *NFKBIA*, *NFKBIB*, *NFKBIE*, *IKBKG*, *NFKBID* and *NFKBIZ* were carried out using a SuperReal PreMix Plus kit (Tiangen Biotech) according to optimized PCR protocols in triplicate, and glyceraldehyde-3-phosphate dehydrogenase (*GAPDH*) was amplified in parallel with the target genes. The primer sequences (Table 1) were designed and synthesized by Shanghai Sangon Biotech Co., Ltd. China. PCR conditions were 40 cycles of 95 °C for 10 s, 60–62.5 °C (60 °C for *BCL-3*, 60.5 °C for *NFKBIA*, *NFKBID* and *NFKBIZ*, 61 °C for *NFKBIB* and *NFKBIE*, 62.5 °C for *IKBKG*) for 20 s and 72 °C for 25 s. Melting curve analysis and electrophoresis on 2% agarose gel were used to verify PCR results. The *GAPDH* gene was used as an internal control, and positive and negative controls were tested every time. The 2^−ΔΔCt^ analysis method [63] was used to calculate relative expression value with *GAPDH* as a normalization control. The data from the ewes on day 16 of the estrous cycle were used as normalization control.

### 4.3. Western Blot

The samples of spleens and lymph nodes were prepared as described previously [7,9], and total proteins were separated on polyacrylamide gels. Total proteins were transferred to PVDF membranes (Millipore, Bedford, MA, USA), and the membranes were incubated with primary antibodies overnight at 4 °C. Membranes were blocked with 5% skimmed milk powder. An anti-BCL-3 monoclonal antibody (Santa Cruz Biotechnology, Santa Cruz, CA, USA, sc-32741), an anti-IκBα monoclonal antibody (Santa Cruz Biotechnology, sc-1643), an anti-IκBβ monoclonal antibody (Santa Cruz Biotechnology, sc-390622), an anti-IκBε monoclonal antibody (Santa Cruz Biotechnology, sc-7275), an anti-IKKγ monoclonal antibody (Santa Cruz Biotechnology, sc-166398), an anti-NFKBID polyclonal antibody (Abcam, Cambridge, UK, ab232913, 1:1000) and an anti-IκBζ polyclonal antibody (Abcam, ab155142, 1:1000) were used. Depending on the origin of the primary antibodies, goat anti-mouse IgG-HRP (Biosharp, Hefei, China BL001A) or goat anti-rabbit IgG-HRP (Biosharp, BL003A) were used in 1:10,000 dilution. After washing, the membranes were incubated with an ECL Western blotting detection reagent (Tiangen Biotech), and the signals were detected. The immunospecific bands were analyzed using the Quantity One 4.1 software (Bio-Rad Laboratories, Hercules, CA, USA) with GAPDH as an internal control protein using an anti-GAPDH antibody (Santa Cruz Biotechnology, Inc., sc-20357, 1:1000).

### 4.4. Immunohistochemistry Analysis

The fixed samples of spleens and lymph nodes were prepared as described previously [7,9]. Several sections were stained by hematoxylin and eosin (HE). Endogenous peroxidase activity of other sections was quenched using 3% H_2_O_2_, and nonspecific binding was reduced with 5% normal goat serum. Immunohistochemical localization of IκBβ and IKKγ in the spleen and lymph nodes was performed using the anti-IκBβ monoclonal antibody (Santa Cruz Biotechnology, sc-390622, 1:200) and anti-IKKγ monoclonal antibody (Santa Cruz Biotechnology, sc-166398, 1:200), and a negative control was treated with goat anti-rabbit IgG. The antibody binding sites in the tissue sections was visualized using a DAB kit (Tiangen Biotech), and then nuclear was stained with hematoxylin. The images were captured using a light microscope (Nikon Eclipse E800, Tokyo, Japan) with a digital camera (AxioCam ERc 5s), and the intensity of staining and density of the stained cells were analyzed through the images as described previously [7,9].

### 4.5. Statistical Analysis

Data for relative expression levels of *BCL-3*, *NFKBIA*, *NFKBIB*, *NFKBIE*, *IKBKG*, *NFKBID* and *NFKBIZ* mRNA and proteins were analyzed with MIXED procedure in SAS (Version 9.1; SAS Institute, Cary, NC, USA). Relative expression levels of the different groups were compared using Duncan’s method, and data are presented as least-squares means. A *p* value < 0.05 was considered significantly different.

## 5. Conclusions

Expression of the IκB family was altered in the maternal spleen and lymph nodes in a tissue-specific manner during early pregnancy, which may be related to the IFNT from conceptus and the progesterone from the corpus lutea. These changes may be associated with establishment of maternal immune tolerance during early pregnancy.

## Figures and Tables

**Table 1:** Primers used for RT-qPCR.

Gene	Primer	Sequence	Size (bp)	Accession Numbers
*BCL-3*	Forward	GCGACCAGAGGCAATTTACTACCAG	98	XM_027978453.2
	Reverse	GAGGTGTAGGCAAGTTCAGCAGAG		
*NFKBIA*	Forward	AGGACGAGGAGTATGAGCAGATGG	130	NM_001166184.1
	Reverse	GCCAAGTGCAGGAACGAGTCTC		
*NFKBIB*	Forward	CCCCAAGACCTACCTCGCTCAG	119	XM_027978262.2
	Reverse	TCCAGTCCTCTTCACTCTCATCCTC		
*NFKBIE*	Forward	GCACTCACGTACATTTCCGAGGAC	97	XM_042236979.1
	Reverse	GCAGCAGAGCCAGGCAATACAG		
*IKBKG*	Forward	GGGCAACCAGAGGGAGGAGAAG	146	XM_027963334.2
	Reverse	GGCATGTCTTCAGGCGTTCCAC		
*NFKBIZ*	Forward	GCAAAGGCGTACAATGGAAACACC	137	NM_001306117.1
	Reverse	GGCTGCTCGTTCTCCAAGTTCC		
*NFKBID*	Forward	ACATTCGTGAGCATAAGGGCAAGAC	114	XM_027977435.2
	Reverse	GATGGTCAGTGGCATTGGGTTCC		
*GAPDH*	Forward	GGGTCATCATCTCTGCACCT	176	NM_001190390.1
	Reverse	GGTCATAAGTCCCTCCACGA		

## Data Availability

Data supporting the findings of this study are available within the paper.
